# Comparison of value of biomarkers in diagnosing lung cancer: An overview of systematic reviews protocol

**DOI:** 10.1097/MD.0000000000015525

**Published:** 2019-05-13

**Authors:** Fanqi Wu, Hong Wang, Hongyan Tao, Huirong Huang, Longguo Zhang, Di Wu, Yixin Wan

**Affiliations:** a Department of Respiratory, Lanzhou University Second Hospital; b The Second Clinical Medical School of Lanzhou University, Lanzhou, China.

**Keywords:** adjusted indirect comparison, biomarker, diagnostic test accuracy, evidence mapping, lung cancer, overview

## Abstract

**Background:**

In both sexes combined, lung cancer is the most commonly diagnosed cancer and the leading cause of cancer death. Furthermore, the incidence rate is increasing in many countries. Many lung cancer patients have a poor prognosis because they are usually diagnosed at an advanced stage. Therefore, there is an urgent need to develop effective methods for early diagnosis of lung cancer. Some systematic reviews have evaluated the value of biomarkers for diagnosing lung cancer. However, it remains unclear which biomarker has superior performance for early and accurate detection of lung cancer. This overview aims to assess the methodological and reporting quality of available systematic reviews and to find an optimal biomarker for diagnosing lung cancer.

**Methods:**

We searched PubMed, Embase.com, the Cochrane Library of Systematic Reviews, and Web of Science to identify relevant systematic reviews including randomized controlled trials, cross-sectional studies, case-control studies, or cohort studies that reported the value of biomarkers for diagnosing lung cancer. The methodological quality will be assessed using AMASAR-2 checklist, and the reporting quality will be assessed using PRISMA-DTA checklist. Bubble plot will be generated to map the biomarkers, methodological and reporting quality. The pairwise meta-analysis and indirect comparisons will be performed using STATA 13.0.

**Results:**

The results of this study will be published in a peer-reviewed journal

**Conclusion:**

This overview will provide comprehensive evidence of different biomarkers for the diagnosis of lung cancer.

**Ethics and dissemination:**

Ethics approval and patient consent are not required as this study is an overview based on published systematic reviews.

## Abstract 2

Supplemental Digital Content is available in the text

## 1 Introduction

Lung cancer is the most commonly diagnosed cancer and the leading cause of cancer death in men. Among women, lung cancer is the third commonly diagnosed cancer and the second cause of cancer death.^[1]^ In 2018, there are an estimated 2.1 million new cases of lung cancer and 1.8 million deaths worldwide, accounting for nearly one-fifth of cancer deaths.^[1]^ Furthermore, the incidence rate is increasing in many countries.^[2,3]^ The 5-year survival rates for patients with advanced lung cancer and metastatic lung cancer were 16.8% and <5%, respectively, while the 5-year survival rate for small intrapulmonary cancer was 80%.^[4–6]^ One of the important reasons for poor prognosis in advanced lung cancer is the lack of effective screening or early diagnostic methods, resulting in many cases missing the best treatment opportunity in clinical diagnosis.^[7–9]^ Therefore, there is an urgent need to develop effective methods for early diagnosis of lung cancer to improve the survival rate of lung cancer patients.^[10,11]^

Traditional pathological biopsy can improve the accuracy of diagnosis, but this invasive surgery can cause great inconvenience and pain to patients.^[12]^ Previous studies have explored imaging methods for the diagnosis of lung cancer, however, these methods either have low sensitivity or low specificity and are therefore not ideal methods for early diagnosis of lung cancer.^[13–16]^ Therefore, many scholars have begun to search for biomarkers with high sensitivity and specificity to diagnose lung cancer, and some biomarkers have shown potential diagnostic value.^[17,18]^

Well-conducted systematic reviews (SRs) and meta-analyses of randomized controlled trials (RCTs) often provide the best evidence for clinical practice and healthcare decisions.^[19–21]^ Recently, some SRs have evaluated the value of biomarkers for diagnosing lung cancer.^[22–24]^ However, the results of these studies are heterogeneous, and it is not clear which biomarker has superior performance for early and accurate detection of lung cancer. The objectives of this overview are to explore the methodological and reporting quality of available SRs, assess diagnostic accuracy of biomarkers for lung cancer, and to compare the diagnostic value of different biomarkers with adjusted indirect comparisons.

## 2 Methods

### 2.1 Design and registration

This protocol will be reported according to preferred reporting items for systematic review and meta-analysis protocols (PRISMA-P).^[25]^ As a part of our project, this protocol has been registered on international prospective register of systematic review (PROSPERO) (CRD42019125880).

### 2.2 Search strategy

The search strategy has been developed and tested through an iterative process by an experienced medical information specialist in consultation with the review team.^[26]^ A combination of subject terms and keywords was used and make appropriate adjustments of vocabulary and grammar between different databases. We searched PubMed, Embase.com, the Cochrane Library of Systematic Reviews, and Web of Science to identify relevant SRs from inception to February 2019. The search was not restricted by language or publication status. Reference lists of relevant SRs will be searched for potentially eligible studies. Study authors will be contacted for methodological clarifications and provision of missing data. The search strategy of EMBASE was presented in Supplementary 1.

### 2.3 Eligibility criteria

Studies will be included in this overview if meet the following eligibility criteria: participants: any patient with lung cancer will be included regardless of the treatment plan and tumor stage. Lung cancer can be non-small cell lung cancer, small cell lung cancer, or other types. There are no restrictions on age, race or nationality. Interventions: All biomarkers used to diagnose lung cancer, including some common tumor biomarkers and some tumor-specific biomarkers. The diagnostic test can be one biomarker or one biomarker combines with other biomarkers. Type of studies: systematic reviews including randomized controlled trials, cross-sectional studies, case-control studies, or cohort studies will be included, as well as the SRs evaluating the value of biomarkers for diagnosing lung cancer. The SRs should report adequate search strategy, inclusion/exclusion criteria, sufficient details about the included studies, the diagnostic value of at least one biomarker. Outcomes: we will consider sensitivity, specificity, positive likelihood ratio, negative likelihood ratio, diagnostic odds ratio, area under the curve, and their respective 95% confidence intervals as the primary outcomes. The relative diagnostic estimates of different biomarkers and the methodological and reporting quality of each SR will be the second outcomes.

### 2.4 Study selection

We will use the EndNote X8 (Thomson Reuters [Scientific] LLC Philadelphia, PA) to manage the retrieved records. The titles and abstracts of the identified studies from the electronic database search will be read by 2 independent reviewers to determine if they meet the inclusion criteria. Then, the same 2 reviewers will retrieve the full text of all possibly relevant studies and assess the eligibility of each study according to the eligibility criteria. To avoid overlapping SRs, we will first map the research questions and characteristics of all eligible SRs. If we identify multiple reviews addressing the same research question that are eligible for inclusion but share the same primary study, we will include the review with the larger number of studies.^[27]^ Different opinions on eligibility for inclusion will be resolved through discussion and consensus. Arbitration will be conducted by a third examiner.

### 2.5 Data extraction and management

Two reviewers will independently extract data, including study characteristics and test results, by using a pre-designed data extraction form. The detailed extracted data will include author, country of corresponding author, number of authors, publication year, journal name, country of journal, funding, disease, number and name of biomarkers, number and name of reference test, types of included studies, number of included studies, samples, pooled sensitivity, specificity, likelihood ratio, diagnostic odds ratio, area under curve, and their 95% confidence interval (95% CI). If there is no specific data in the published SRs, the author will be contacted to provide the missing information. Disagreements will be resolved by consensus or by discussion with a third reviewer.

### 2.6 Assessment of methodological and reporting quality

Assessment of Multiple Systematic Reviews (AMSTAR), a reliable methodological quality assessment tool for SRs of randomized trials, has a good agreement, construct validity, and feasibility.^[28–30]^ AMSTAR-2 is a revised revision of the original AMSTAR instrument and can be used to evaluate the quality of SRs based on non-RCTs.^[31,32]^ The Preferred Reporting Items for Systematic Reviews and Meta-analysis diagnostic test accuracy (PRISMA-DTA) is an expanded checklist of original PRISMA, which aims to improve the completeness and transparency of reporting of SRs of diagnostic test accuracy studies.^[33]^ Thus, the AMSTAR-2 will be used to assess the methodological quality and the PRISMA-DTA for reporting quality of included SRs. Two review authors will independently assess the quality in each study according to predefined criteria. Disagreements will be resolved by consensus or third-party adjudication if consensus cannot be reached.

### 2.7 Data synthesis

#### 2.7.1 Evidence map

We will create a bubble plot according to the biomarkers, methodological and the reporting quality for all included SRs using R 3.4.1 software (version 3.4.1; R Foundation for Statistical Computing, Vienna, Austria). Each bubble plot will display information in 3 dimensions. The bubble size represents the total number of reviews or the number of primary studies included in the SRs. The *x*-axis will represent the total number of participants included in each SR or the methodological quality. The *y*-axis will represent the biomarkers or the reporting quality of each SR.

#### 2.7.2 Pairwise meta-analysis

Pairwise meta-analysis will be performed for pooled sensitivity, specificity, DOR, positive likelihood ratio, negative likelihood ratio using the Mantel-Haenszel statistical method with the random-effects model with STATA (13.0; Stata Corporation, College Station, TX). The heterogeneity between each study will be estimated using the *P* value and the *I*^2^ statistic. The values of 25%, 50%, and 75% for the *I*^2^ will be indicative of low, moderate, and high statistical heterogeneity, respectively.^[34]^

#### 2.7.3 Adjusted indirect comparisons

We will calculate relative diagnostic outcomes between index tests including relative sensitivity, relative specificity, and relative DOR. Then, we will conduct indirect comparisons using relative diagnostic outcomes.

#### 2.7.4 Assessment of reporting bias

The Begg test will be used to evaluate the potential publication bias where there are >10 SRs available for a biomarker.

#### 2.7.5 Subgroup analysis

Subgroup analysis will be performed according to the types of lung cancer, the country in which the study was conducted, and the cutoff and time period of biomarkers if sufficient data are available.

## Author contributions

Fanqi Wu and Yixin Wan planned and designed the research; Hongyan Tao, Huirong Huang, Longguo Zhang, and Di Wu tested the feasibility of the study; Fanqi Wu and Yixin Wan wrote the manuscript; all authors approved the final version of the manuscript.

**Conceptualization:** Fanqi Wu, Yixin Wan.

**Data curation:** Fanqi Wu, Hong Wang, Huirong Huang, Longguo Zhang, Di Wu.

**Funding acquisition:** Fanqi Wu.

**Investigation:** Hong Wang, Hongyan Tao, Huirong Huang, Longguo Zhang, Di Wu.

**Methodology:** Yixin Wan.

**Project administration:** Fanqi Wu.

**Resources:** Hong Wang, Hongyan Tao, Huirong Huang.

**Software:** Longguo Zhang, Di Wu.

**Supervision:** Yixin Wan.

**Validation:** Yixin Wan.

**Visualization:** Hongyan Tao.

**Writing – original draft:** Fanqi Wu, Yixin Wan.

**Writing – review & editing:** Fanqi Wu, Yixin Wan.

## Supplementary Material

Supplemental Digital Content
