# Supplementary material for: Comparison of value of biomarkers in diagnosing lung cancer: An overview of systematic reviews protocol
Source: Medicine (Baltimore). 2019 May 13;98(19):e15525. doi: 10.1097/MD.0000000000015525 (PMC6531075; doi:10.1097/MD.0000000000015525)
Supplement: Supplemental Digital Content [file medi-98-e15525-s001.docx]

**The search strategy of EMBASE**

#1 'lung tumor'/exp OR 'lung adenocarcinoma'/exp OR 'lung alveolus cell carcinoma'/exp OR 'bronchus tumor'/exp OR 'lung carcinoma'/exp OR 'multiple pulmonary nodules'/exp OR 'Pancoast tumor'/exp OR 'lung blastoma'/exp OR 'pulmonary sclerosing hemangioma'/exp

#2'lung neoplasm*':ab,ti OR 'lung tumor*':ab,ti OR 'lung carcinoma*':ab,ti OR 'lung cancer*':ab,ti OR 'lung tumour*':ab,ti OR ' pulmonary neoplasm*':ab,ti OR 'pulmonary tumor*':ab,ti OR 'pulmonary carcinoma*':ab,ti OR 'pulmonary cancer*':ab,ti OR 'pulmonary tumour*':ab,ti OR 'bronchial neoplasm*':ab,ti OR 'bronchogenic carcinoma*':ab,ti OR 'bronchial carcinoma*':ab,ti OR 'lung adenocarcinoma*':ab,ti OR 'alveolar adenocarcinoma*':ab,ti OR 'alveolar carcinoma*':ab,ti OR 'alveolar cell carcinoma*':ab,ti OR 'bronchiolar carcinoma*':ab,ti OR 'bronchioloalveolar carcinoma*':ab,ti OR 'pancoast syndrome':ab,ti OR 'pulmonary sclerosing hemangioma*':ab,ti OR 'lung sclerosing hemangioma*':ab,ti OR 'multiple pulmonary nodule':ab,ti OR 'non-small-cell lung carcinoma*':ab,ti OR 'nonsmall cell lung cancer*':ab,ti OR 'non small cell lung carcinoma*':ab,ti OR 'non-small cell lung cancer*':ab,ti OR 'small cell lung cancer*':ab,ti OR 'oat cell lung cancer*':ab,ti

#3 #1 OR #2

#4 'tumor marker'/exp OR 'biological marker'/exp

#5 'cancer biomarker*':ab,ti OR 'cancer bio-marker':ab,ti OR 'cancer marker*':ab,ti OR 'tumor biomarker*':ab,ti OR 'tumor bio-marker':ab,ti OR 'tumor marker*':ab,ti OR 'tumour biomarker*':ab,ti OR 'tumour bio-marker':ab,ti OR 'tumour marker*':ab,ti OR 'carcinogen biomarker*':ab,ti OR 'carcinogen bio-marker':ab,ti OR 'carcinogen marker*':ab,ti OR 'neoplasm biomarker*':ab,ti OR 'neoplasm bio-marker':ab,ti OR 'neoplasm marker*':ab,ti OR 'neoplasm metabolite marker*':ab,ti OR 'tumor metabolite marker*':ab,ti OR 'cancer metabolite marker*':ab,ti OR 'tumour metabolite marker*':ab,ti OR 'carcinogen metabolite marker*':ab,ti

#6 #4 OR #5

#7 'Sensitivity AND Specificity'/exp OR 'False Positive Reactions'/exp OR 'False Negative Reactions'/exp OR 'ROC Curve'/exp OR 'Predictive Value of Tests'/exp

#8 'sensitivity':ab,ti OR 'specificity':ab,ti OR 'receiver operating characteristic':ab,ti OR 'receiver operator characteristic':ab,ti OR 'predictive value*':ab,ti OR 'roc':ab,ti OR 'pre-test odds':ab,ti OR 'pretest odds':ab,ti OR 'pre-test probability*':ab,ti OR 'pretest probability*':ab,ti OR 'post-test odds':ab,ti OR 'posttest odds':ab,ti OR 'post-test probabilit*':ab,ti OR 'posttest probabilit*':ab,ti OR 'likelihood ratio*':ab,ti OR 'positive predictive value*':ab,ti OR 'negative predictive value*':ab,ti OR 'false negative*':ab,ti OR 'false positive*':ab,ti OR 'true negative*':ab,ti OR 'true positive*':ab,ti

#9 #7 OR #8

#10 'meta analysis (topic)'/exp OR 'meta analysis'/exp OR 'systematic review'/exp OR 'systematic review (topic)'/exp

#11 'meta analysis':ab,ti OR 'meta analyses':ab,ti OR 'meta-analysis':ab,ti OR 'meta-analyses':ab,ti OR 'metaanalysis':ab,ti OR 'metanalysis':ab,ti OR 'met-analysis':ab,ti OR 'metaanalyses':ab,ti OR 'metanalyses':ab,ti OR 'met-analyses':ab,ti OR 'data pooling':ab,ti OR 'data poolings':ab,ti OR 'clinical trial overview':ab,ti OR 'clinical trial overviews':ab,ti OR 'systematic review':ab,ti OR 'systematic reviews':ab,ti

#12 #10 OR #11

#13 #3 AND #6 AND #9 AND #12
